# P-1785. Characterizing the epidemiological and clinical diversity of Nipah virus strains from Bangladesh: 2016 to 2023

**DOI:** 10.1093/ofid/ofaf695.1954

**Published:** 2026-01-11

**Authors:** Syed Moinuddin Satter, Dewan Rahman, Mohammed Ziaur Rahman, Sharmin Sultana, Md Mahfuzur Rahman, Wasik Rahman Aquib, Arifa Nazneen, Anika Farzin, Kamal Ibne Amin Chowdhury, Tonmoy Sarkar, Fatema Akther Ema, Shadman Sakib Choudhury, Ayesha Siddika, Muhammad Rashedul Alam, Faruq Abdulla, Probir Kumar Ghosh, Md Omar Qayum, Md Ferdous Rahman Sarker, Md Abdullah Omar Nasif, Barnali Sen, Mintu Chowdhury, Md Sazzad Hossain, Mahbubur Rahman, Ahmed Nawsher Alam, Mohammad Enayet Hossain, Trevor Shoemaker, Christina Spiropoulou, Emily S Gurley, Stephen Luby, Jhon D Klena, Sayera Banu, Joel M Montgomery, Tahmina Shirin

**Affiliations:** icddr,b (International Centre for Diarrhoeal Disease Research, Bangladesh), Dhaka, Dhaka, Bangladesh; icddr,b, Dhaka, Dhaka, Bangladesh; icddr,b (International Centre for Diarrhoeal Disease Research, Bangladesh), Dhaka, Dhaka, Bangladesh; Institute of Epidemiology, Disease Control and Research (IEDCR), Dhaka, Dhaka, Bangladesh; icddr,b (International Centre for Diarrhoeal Disease Research, Bangladesh), Dhaka, Dhaka, Bangladesh; icddr,b (International Centre for Diarrhoeal Disease Research, Bangladesh), Dhaka, Dhaka, Bangladesh; icddr,b, Dhaka, Dhaka, Bangladesh; icddr,b (International Centre for Diarrhoeal Disease Research, Bangladesh), Dhaka, Dhaka, Bangladesh; icddr,b, Dhaka, Dhaka, Bangladesh; icddr,b (International Centre for Diarrhoeal Disease Research, Bangladesh), Dhaka, Dhaka, Bangladesh; icddr,b, Dhaka, Dhaka, Bangladesh; icddr,b, Dhaka, Dhaka, Bangladesh; icddr,b (International Centre for Diarrhoeal Disease Research, Bangladesh), Dhaka, Dhaka, Bangladesh; icddr,b (International Centre for Diarrhoeal Disease Research, Bangladesh), Dhaka, Dhaka, Bangladesh; icddr,b, Dhaka, Dhaka, Bangladesh; icddr,b (International Centre for Diarrhoeal Disease Research), Shantinagar, Dhaka, Bangladesh; Institute of Epidemiology, Disease Control & Research (IEDCR), Dhaka, Dhaka, Bangladesh; Institute of Epidemiology, Disease Control & Research (IEDCR), Dhaka, Dhaka, Bangladesh; Institute of Epidemiology, Disease Control & Research (IEDCR), Dhaka, Dhaka, Bangladesh; Institute of Epidemiology, Disease Control & Research (IEDCR), Dhaka, Dhaka, Bangladesh; Institute of Epidemiology, Disease Control & Research (IEDCR), Dhaka, Dhaka, Bangladesh; Institute of Epidemiology, Disease Control & Research (IEDCR), Dhaka, Dhaka, Bangladesh; Institute of Epidemiology, Disease Control & Research (IEDCR), Dhaka, Dhaka, Bangladesh; Institute of Epidemiology, Disease Control & Research (IEDCR), Dhaka, Dhaka, Bangladesh; icddr,b (International Centre for Diarrhoeal Disease Research, Bangladesh), Dhaka, Dhaka, Bangladesh; Centers for Disease Control and Prevention (CDC), Atlanta, Georgia; US CDC, Atlanta, Georgia; Johns Hopkins, Baltimore, MD; Stanford University, Palo Alto, California; CDC, Sierra Leone, Greater Accra, Ghana; icddr,b (International Centre for Diarrhoeal Disease Research, Bangladesh), Dhaka, Dhaka, Bangladesh; Centers for Disease Control and Prevention (CDC), Atlanta, Georgia; Institute of Epidemiology, Disease Control and Research (IEDCR), Dhaka, Dhaka, Bangladesh

## Abstract

**Background:**

Nipah virus (NiV), a zoonotic pathogen, is responsible for fatal outbreaks in Bangladesh, with a case fatality rate up to 71%. NiV-BD 1 and NiV-BD 2 are the two sublineages described in human cases in Bangladesh. This study aims to characterize the epidemiological and clinical diversity of NiV strains circulating in Bangladesh.

**Figure:**
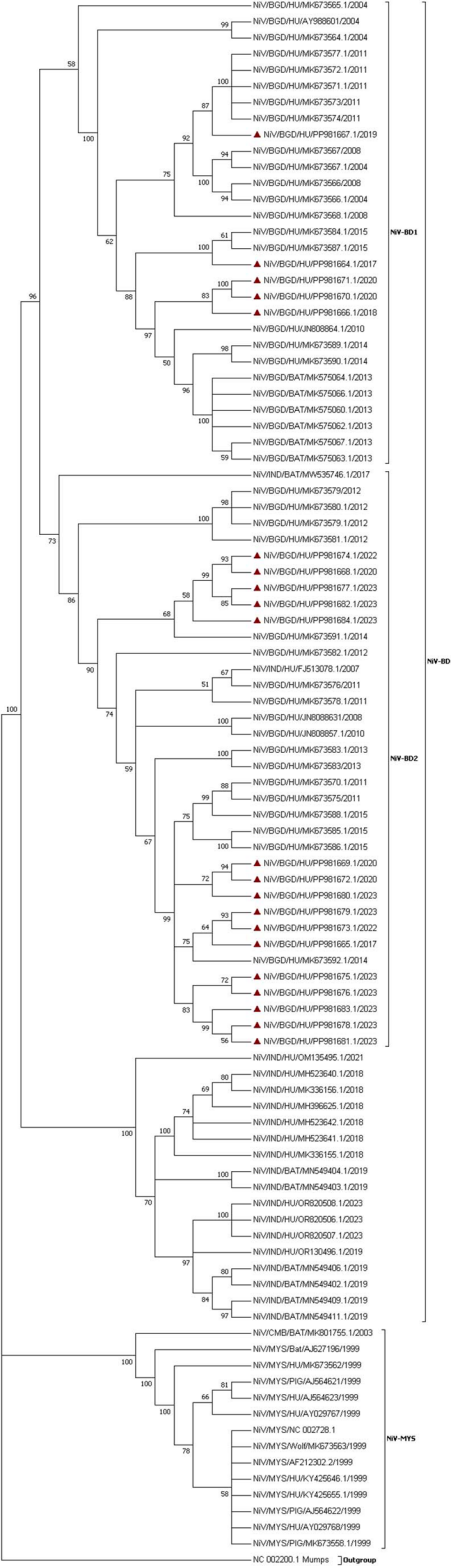
Phylogenic analysis of the Nipah virus genome sequences

**Methods:**

This study combined 21 new human NiV near-complete or partial genome sequences from the period 2016-2023 with 17 previously reported sequences from NiV cases from 2012-2015. The differences in epidemiological and clinical features between the sublineages were compared using descriptive statistics and bivariate analysis.

**Results:**

The median age of the NiV cases examined in this study was 17 years [IQR: 9-30]; males were predominant (66%). Consumption of contaminated raw date palm sap was the primary route of transmission (92%). NiV-BD 1 strain was primarily localized in the northern and central regions of Bangladesh, while NiV-BD 2 showed a broader geographic distribution, with cases also reported in the southern region. The two sublineages did not differ much. No significant difference between age, sex, or transmission modes was observed among those individuals infected with NiV-BD 1 or 2. Clinical symptoms such as fever, altered mental status, and unconsciousness were observed in both strains, while respiratory distress was more prevalent (23/29 cases) in cases infected with NiV-BD 2. Duration of hospitalization was longer than 3 days (IQR: 1-23) in cases infected with NiV-BD 1. The overall mortality rate was 84%, irrespective of sublineage infection.

**Conclusion:**

This study highlights that NiV sublineage vary depending on geographical location and in some of the clinical presentations for which they are responsible. These points will be important when selecting vaccine candidates to ensure that they are protective against both sublineages circulating in Bangladesh.

**Disclosures:**

All Authors: No reported disclosures

